# Medical and Paramedical Care of Patients With Cerebellar Ataxia During the COVID-19 Outbreak: Seven Practical Recommendations of the COVID 19 Cerebellum Task Force

**DOI:** 10.3389/fneur.2020.00516

**Published:** 2020-05-22

**Authors:** Mario Manto, Nicolas Dupre, Marios Hadjivassiliou, Elan D. Louis, Hiroshi Mitoma, Marco Molinari, Aasef G. Shaikh, Bing-Wen Soong, Michael Strupp, Frank Van Overwalle, Jeremy D. Schmahmann

**Affiliations:** ^1^CHU-Charleroi and Service des Neurosciences, University of Mons, Mons, Belgium; ^2^Department of Medicine, Faculty of Medicine, Université Laval & Neuroscience Axis, CHU de Québec-Université Laval, Quebec City, QC, Canada; ^3^Academic Department of Neurosciences, Sheffield Teaching Hospitals NHS Trust, Sheffield, United Kingdom; ^4^Department of Neurology and Neurotherapeutics, University of Texas Southwestern, Dallas, TX, United States; ^5^Department of Medical Education, Tokyo Medical University, Tokyo, Japan; ^6^IRCCS Fondazione S. Lucia, Rome, Italy; ^7^Department of Neurology, Case Western Reserve University, University Hospitals Cleveland Medical Center, Cleveland VA Medical Center, Cleveland, OH, United States; ^8^Taipei Neuroscience Institute, Taipei Medical University, Taipei, Taiwan; ^9^Department of Neurology, Taipei Medical University-Shuang Ho Hospital, New Taipei City, Taiwan; ^10^Department of Neurology, Taipei Veterans General Hospital, and Brain Research Center, National Yang-Ming University, Taipei, Taiwan; ^11^Department of Neurology, Ludwig Maximilians University, Munich, Germany; ^12^Brain, Body and Cognition Lab, Faculty of Psychology, Vrije Universiteit Brussel, Brussels, Belgium; ^13^Ataxia Center, Cognitive Behavioral Neurology Unit, Laboratory for Neuroanatomy and Cerebellar Neurobiology, Department of Neurology, Massachusetts General Hospital and Harvard Medical School, Boston, MA, United States

**Keywords:** SARS-CoV-2, COVID-19, cerebellum, therapies, ataxia

## Abstract

The severe acute respiratory syndrome coronavirus 2 (SARS-CoV2), the cause of the current pandemic coronavirus disease 2019 (COVID-19), primarily targets the respiratory system. Some patients also experience neurological signs and symptoms ranging from anosmia, ageusia, headache, nausea, and vomiting to confusion, encephalitis, and stroke. Approximately 36% of those with severe COVID-19 experience neurological complications. The virus may enter the central nervous system through the olfactory nerve in the nasal cavity and damage neurons in the brainstem nuclei involved in the regulation of respiration. Patients with cerebellar ataxia (CA) are particularly vulnerable to severe outcome if they contract COVID-19 because of the complexity of their disease, the presence of comorbidities, and their use of immunosuppressive therapies. Most CA patients burdened by progressive neurologic deficits have substantially impaired mobility and other essential functions, for which they rely heavily on ambulatory services, including rehabilitation and psychosocial care. Cessation of these interventions because of isolation restrictions places the CA patient population at risk of further deterioration. This international panel of ataxia experts provides recommendations for neurologists caring for patients with CA, emphasizing a pro-active approach designed to maintain their autonomy and well-being: continue long-term medications, promote rehabilitation efforts, utilize the technology of virtual visits for regular contact with healthcare providers, and pay attention to emotional and psychosocial health. Neurologists should play an active role in decision-making in those CA cases requiring escalation to intensive care and resuscitation. Multi-disciplinary collaboration between care teams is always important, and never more so than in the context of the current pandemic.

The novel zoonotic corona virus (severe acute respiratory syndrome coronavirus 2: SARS-CoV2) has caused a global pandemic, coronavirus disease-2019 (COVID-19), which presents primarily with severe pulmonary disease, acute respiratory collapse, and multisystem failure (1). As many as 36% of hospitalized patients in the Chinese experience developed central and peripheral nervous system involvement, including cerebellar manifestations (2). SARS-CoV2 is thought to enter the central nervous system via the bloodstream or by retrograde neuronal transmission through the cribriform plate. It binds to the membrane-bound form of angiotensin-converting enzyme 2 (ACE2) in bronchial alveoli, with internalization of the complex by the host cells (3), the same receptor identified in SARS-CoV, which also affected nervous system function (4).

Cerebellar ataxias (CAs) are a group of heterogeneous disorders from both the phenotypic and genetic standpoints. CAs include chronic neurodegenerative disorders such as the autosomal dominant spinocerebellar ataxias (SCAs), which affect cerebellar and extra-cerebellar structures, including brainstem and motor neurons, with widespread dysfunction of the motor system and other neurological domains. Patients with the various CAs are particularly vulnerable to COVID-19 for several reasons: their neurological syndrome is complex, comorbid medical illnesses are common, immune-mediated ataxias are managed with immunosuppressive medications such as rituximab, mycophenolate, and corticosteroids, which require frequent monitoring, CAs may affect elderly people, and all of these predispositions to severe response to the viral infection are compounded by cerebellum-specific neuropsychological impairments. General practitioners (GPs), who most frequently care for these patients and their families, are reporting that they are overwhelmed by the pandemic, with their usual meticulous engagement in managing their long-term course and supervening acute issues being superseded by the immediate need to care for the surging numbers of COVID-19 patients. Patients with CA in many centers are even finding that they are having difficulty connecting with their suddenly overburdened GPs. Adding to the stress on the CA patient population world-wide is the reality that in-person or at-home support services, including psychological counseling or therapy visits, have stopped, and necessary ambulatory care services such as speech, physical, and occupational therapy interventions have come to an abrupt halt. The consequence is that patients are reporting dramatically decreased levels of rehabilitative and maintenance care, which is having a demonstrable negative impact on their overall care and well-being.

All societies around the world, and most particularly the vulnerable populations, are severely challenged by the COVID-19 pandemic. There are, however, three considerations specific to patients and families dealing with CAs.

First, dysphagia, ataxic respiration, maintenance of airway protection, and aspiration pneumonia are an ever-present concern and risk in CAs (5). Should CA patients contract COVID-19, they are potentially at increased risk for pulmonary complications because dyspnea, cough, and fever, together with systemic ill-health and severe fatigue are among the earliest manifestations.

Second, there is intense pressure on healthcare systems due to limited numbers of beds in intensive care units (ICUs), which are facing an enormous challenge in terms of abrupt reorganization of their procedures and decisions to treat patients (6). Patients with CA may be at high risk now because our ICU colleagues might consider patients with CA to have irreversible neurological diseases with no hope of cure and who should therefore not benefit from ICU-level care. Genetic ataxias, such as Friedreich's ataxia (FA) with its cardiomyopathy and diabetes, represent a typical example of a neurological disorder with poor prognosis according to colleagues in ICUs. But not all CA patients are the same: a patient with late-stage FA with cardiomyopathy and diabetes is quite different than a young, otherwise medically healthy patient with FA or a late-onset adult FA patient whose disease resembles a more slowly evolving spinocerebellar ataxia. The same concept is true when distinguishing between a patient with multiple system atrophy of the cerebellar type and a patient with spinocerebellar ataxia type 6. Nuances of knowledge about the nature of the underlying disease of a CA patient are second-nature to neurologists and particularly to ataxiologists, but they are not necessarily familiar to internists, who need input and guidance from the neurologist expert in these disorders.

Third, the neurocognitive and behavioral-affective syndrome in CA patients manifests with impairments in executive function and visuo-spatial cognition and in personality changes, including blunting of affect or impaired behavior (7). Patients are at risk for severe depression and apathy, as well as reduced mental flexibility (8). Impulsive actions, propensity to rumination, and unawareness of social boundaries are not unexpected in some of the CAs. Impaired attentional control combined with illogical thought impacts daily life, with the consequence that patients with CA patients occupy the borderland of neurology and neuropsychiatry and require specialized management (9).

In our institutions, we have instituted the following seven practices and share these recommendations with neurologists caring for the CA patient population:

1. Emphasize that the recommendations to the general population to avoid contracting the infection need to be followed seriously and rigorously by CA patients, their families, and caregivers. This includes staying at home as long as the local/national authorities determine quarantine is still required, maintaining physical distancing if leaving the house or interacting with others outside the immediate household, adhering to the advertised protocols for hand-washing, avoiding touching the face, wearing masks when in contact with others outside the home, and meticulous cleansing and disinfecting of surfaces and of objects brought into the house. Take a pro-active approach to educating patients regarding travel restrictions.
2. Continue all necessary medications, whether for the symptoms and manifestation of the CA or for other medical conditions, for instance, 4-aminopyridine or acetazolamide in episodic ataxias. Patients with immune-mediated ataxias should not discontinue their immunosuppressive drugs. The dysphoria, anhedonia, and sometimes suboptimal decision-making encountered in CA patients may contribute to unjustified interruption of medications or undue influence by social media sites offering unsolicited and erroneous medical advice.
3. Promote rehabilitation efforts through speech and language therapy and physical and occupational therapy in CA patients. Discontinuation of rehabilitation is likely to exacerbate symptoms and worsen ataxia, as is already evident in our phone or video virtual visits with patients. Contact should be maintained with providers of these rehabilitation services to devise programs that can be carried out at home and monitored remotely. The effect of this cessation of in-person rehabilitation care provides an opportunity for quantitative research in the future to assess the impact of this unexpected change in the care model. Because neurological disorders are not uncommon in COVID-19, it may be relevant to remind patients, caregivers, and paramedics that the ataxic syndrome may worsen due to the lack of rehabilitation or the interruption of medications. This exacerbation should be distinguished from a new-onset COVID-19 infection, which is primarily characterized by fever, dry cough, tiredness, pains, nasal congestion, sore throat, or diarrhea.
4. Use the virtual visit platforms of telemedicine. We are fortunate to have this technology, which has emerged as the primary way to interact with and care for patients through telephone contact or real-time interactive video platforms in this environment in which in-person encounters for routine care are not possible. Whereas, telemedicine technology is not perfect or equipped to perform a comprehensive neurological examination, in our experience it is nevertheless adequate for assessing the overall mental state and speech and for examination of hyperkinetic movement disorders, ataxia, hand dexterity, and balance, while maintaining the social connection with patients and their families and giving them the confidence that they are cared for and are not being abandoned.
5. Emphasize the need for assessment and monitoring of patients' emotional and social health, which may be adversely affected in this time of great stress and anxiety and superimposed upon the real social isolation that many CA patients already feel because of the nature of their underlying disorder. Be aware that emotional and social impairments of cerebellar patients may render virtual interaction more difficult and taxing, so that extra encouragement might be needed to use virtual means. These patients may also have diminished understanding of the social duty to follow the strict hygiene measures necessary for the common welfare (10). The cognitive and affective deficits in some CA patients may prevent them from identifying or reporting the possible symptoms of the novel coronavirus infection. This is an additional reason to recommend that CA patients maintain frequent contact with families and caregivers, using the technology of virtual interactions if necessary (see also recommendation 4).
6. Take an active role in management decisions regarding CA patients stricken with COVID-19 whose illness may require ICU-level care in the context of reduced access to ventilator care. Current management strategies need to be fine-tuned in these instances (11). Internists and intensivists can benefit from the neurologist's understanding and experience of the preexisting CA condition and its implications, and the neurologist can advocate for these patients. Careful neurological examinations are also recommended, keeping in mind that coronavirus invasion in the brain may not trigger the classical inflammation cascade leading to the usual picture of infectious encephalitis (12). Nobody is better positioned than the neurologist to ascertain the neurological prognosis of CA patients. Collaborative thinking across disciplines should be promoted when facing ethical challenges that may arise in caring for patients with CA. In case of suspected or confirmed COVID-19 in CA patients with more severe manifestations, both the motor and non-motor features of the cerebellar syndrome need to be considered during quarantine or in the isolation room. Patients are typically clumsy and may require adaptation of the environment, for instance, access to food. They also require a case-by-case discussion for the management of neuropsychiatric manifestations. For CA patients with COVID-19 whose illness may require ICU-level care and intubation, consideration of hospice-level care may be appropriate if the underlying CA is severe and has a short life-expectancy. This decision poses its own challenges, as hospices may lack staff and equipment. Assistance choices for CA patients who are already in bad condition with short life-expectancy and stricken with COVID-19 requiring ICU-level care and intubation are ethically difficult. On one side, clinical pre-COVID-19 conditions must not preclude, a-priori, access to ICU. On the other, in case of limited resources, as is often the case in pandemic conditions, disaster medicine rules apply.
7. Be mindful in this unprecedented time that it is fully appropriate to be attuned to the needs of our medical and paramedical colleagues who are themselves in need of attention (13). Insomnia, anxiety, depression, and even suicide are now reported in healthcare providers exhausted by the workload and fearful of succumbing to the disease themselves. An antidote to the emotional, intellectual, and physical depletion that is characterizing this global crisis is the international and multi-disciplinary discussion and collaboration focused on saving lives and advancing science, and we all play a role in the effort to emerge stronger once this scourge has been defeated.

None of us has ever personally encountered such a dire situation as is posed by this pandemic. This set of recommendations offered by the international panel of ataxia experts is based on our collective clinical experience and review of the rapidly expanding clinical and basic science literature. We expect that the results of clinical trials and larger studies of the pandemic in all its manifestations will enable us to provide more substantive and evidence-based guidelines for neurologists, including ataxiologists, in the future.

## Author Contributions

All authors listed have made a substantial, direct and intellectual contribution to the work, and approved it for publication.

## Conflict of Interest

The authors declare that the research was conducted in the absence of any commercial or financial relationships that could be construed as a potential conflict of interest.
